# Efficacy and safety profile of COVID-19 mRNA vaccine in patients with hematological malignancies: Systematic review and meta-analysis

**DOI:** 10.3389/fonc.2022.951215

**Published:** 2022-08-05

**Authors:** Ikhwan Rinaldi, Samuel Pratama, Lowilius Wiyono, Jeremy Rafael Tandaju, Indy Larasati Wardhana, Kevin Winston

**Affiliations:** ^1^ Hematology and Medical Oncology Division, Department of Internal Medicine, Cipto Mangunkusumo National General Hospital, Jakarta, Indonesia; ^2^ Faculty of Medicine, University of Indonesia, Jakarta, Indonesia; ^3^ Hospital Medicine, Bhakti Medicare Hospital, Cicurug, Sukabumi, Indonesia

**Keywords:** COVID-19, mRNA vaccine, hematologic malignancies, seroconversion rates, antibody titers, adverse effects

## Abstract

Patient populations, including those with hematological malignancies, have different responses to COVID-19 vaccines. This study aimed to quantitatively analyze the efficacy and safety of COVID-19 mRNA vaccines in patients with hematological malignancies. Studies reporting on the efficacy and safety of COVID-19 mRNA vaccines in cohorts with hematological malignancies compared to healthy controls were systematically searched in four databases. Meta-analysis and subgroup analyses were performed to generate quantitative synthesis. Fifteen studies with 2,055 cohorts with hematological malignancies and 1,105 healthy subjects as control were included. After two doses of COVID-19 vaccination, only 60% of cohorts with hematological malignancies were seroconverted compared to healthy controls (RR 0.60; 95%CI 0.50–0.71). A single dose of the vaccine resulted in a significantly lower seroconversion rate (RR 0.30; 95%CI 0.16–0.54). Non-Hodgkin lymphoma cohorts had the lowest rate of seroconversion (RR 0.5; 95%CI 0.35–0.71) and those who received active treatments had lower immunological responses (RR 0.59; 95%CI 0.46–0.75). Antibody titers were lower in cohorts with hematological malignancies without any differences in adverse effects in both groups. In conclusion, cohorts with hematological malignancies showed a lower seroconversion rate and antibody titers after receiving COVID-19 mRNA vaccines. The type of malignancy and the status of treatment had a significant impact on the response to vaccination. The vaccines were shown to be safe for both patients with hematological malignancies and healthy controls. Booster doses and stricter health protocols might be beneficial for patient populations.

## Introduction

The severe acute respiratory syndrome coronavirus 2 (SARS-CoV-2) virus has spread worldwide since its first case in 2019, with 500 million infected people with coronavirus disease 2019 (COVID-19) and more than 6.1 million deaths (1). The virus has caused great difficulty across the globe, compromising service in healthcare facilities and in different public sectors. Actions towards managing the pandemic have been implemented, starting with social distancing measures in numerous countries. Social distancing itself has been associated with reduction of COVID-19 incidence by 29% and a 35% reduction of COVID-19 mortality, and even almost reached zero cases in China in 2020, prior to the emergence of cases due to new mutant variants (2, 3). The vaccination program, started in 2020, has been considered the key approach for ending the pandemic. In total, more than 11 billion doses have been administered with around 59% of the world’s population fully vaccinated (4). A total of 46 trials have been conducted on the COVID-19 vaccine, from messenger ribonucleic acid (mRNA) vaccines, inactivated vaccines, protein subunit vaccines, to viral vector vaccines (5). The vaccination program has reported a good outcome, with an efficacy of 61%–95%, thus decreasing the mortality rate, the incidence of severe diseases and the incidence of infection to almost 90% after the implementation of the vaccination program, despite growing concerns of short-term immunity, especially concerning the mRNA vaccines (6–9).

Despite the success of COVID-19 vaccines with its high efficacy on the healthy population, concerns on the efficacy and safety of COVID-19 vaccines in the immunocompromised population remain unresolved. The seroconversion rate has been found to be significantly lower in immunocompromised patients, especially organ transplant recipients, with only one third achieving seroconversion status, followed by patients with immune-mediated inflammatory disorders (75%), and malignancies or cancers ranging from 63% –90% achieving seroconversion (7, 10). The same concern is also directed at patients with hematological malignancies, especially due to their high risk of being immunosuppressed due to the pathogenesis and molecular mechanism of the disease. One study has mentioned the lower positive seropositivity rate ranging from 62%–66% in patients with hematological malignancies, those with B-cell malignancies, and those undergoing active monoclonal antibody treatment (11). The response rate of COVID-19 vaccines in hematological malignancies is also lower than the solid cancer group by almost two fold (12). RNA vaccines are considered the vaccine with the highest efficacy, despite their short-term immunogenicity. The BNT162b2 and mRNA-1273 have been shown to be 95.3% and 84.4% effective, respectively, in healthy populations (13). Therefore, the mRNA vaccine could be suitable as an option for immunosuppressed patients (6). Previous cumulative data on the efficacy and safety of mRNA vaccines have been collected in immunosuppressed patients (14), and in patients with hematological malignancies by Cavanna et al. (15) and Sakuraba et al. (10). However, due to the small number of included studies and the wide confidence interval, the results might seem insignificant. Thus, this systematic review and meta-analysis was conducted to evaluate the efficacy of mRNA vaccines on hematological malignancies to include the most recent studies on the topic.

## Methods

This study was conducted in accordance with Preferred Reporting Items for Systematic Review and Meta-Analysis (PRISMA) guidelines (16). This systematic review was also subject to the Assessing the Methodological Quality of Systematic Reviews (AMSTAR) 2 checklist (17).

### Search protocol registration

The protocol of this study was registered in the PROSPERO database (CRD42022316188).

### Ethical approval

This systematic review used studies and the grey literature published in several medical databases. Ethics approval was not required for this study.

### Search strategy

Studies related to the efficacy and safety of the COVID-19 mRNA vaccine in cohorts with hematological malignancy were systematically searched in databases including PubMed, Scopus, ScienceDirect, and EBSCOHost. General search terms used included: ‘COVID-19’, ‘SARS-CoV-2’, ‘nCOV-19’, ‘mRNA vaccine’, ‘BNT162’, ‘mRNA-1273’, ‘malignancy’, ‘neoplasm’, and ‘cancer’. The search was carried out on March 15, 2022 with detailed keywords used for each database, as shown in **Table 1**. The search was limited to literature in English, but no limitation was used on the publication period. Manual searching of references was used to identify additional suitable studies. Duplicate entries were then removed, followed by screening of titles and abstracts. Full-text reviews were conducted to identify potentially eligible titles and abstracts by each investigator. The search and screening were conducted independently by five investigators (IR, IW, JR, LW, and SP). Reasons for excluding studies were listed.

**Table 1:** Literature was queried in four databases using the following keywords.

Database	Keywords
PubMed	((“mRNA Vaccines”[Mesh]) AND “COVID-19 Vaccines”[Mesh]) AND (“Neoplasms”[Mesh]))
ScienceDirect	(“mRNA” AND “vaccine”) AND (“neoplasm” OR “malignancy” OR “tumor” OR “cancer”) AND (“SARS-CoV-2” OR “COVID”)
EBSCOHost	((MH “COVID-19 Vaccines+”) OR (MH “2019-nCoV Vaccine mRNA-1273”) OR (MH “BNT162 Vaccine”)) AND ((“Malignancy” OR “neoplasm”))
SCOPUS	(“COVID-19” OR “nCOV-19” OR “Coronavirus disease 2019” OR “SARS-COV-19”) AND (“mRNA vaccine” OR “mRNA-1273” OR “BNT162”) AND (“malignancy” OR “neoplasm”)

### Study eligibility criteria

The following criteria were used to select the studies to be included in the analysis: 1) prospective cohort studies or clinical trials of COVID-19 vaccines in cohorts with hematological malignancies or sub-analysis of hematological malignancies, 2) vaccination with any formulation of COVID-19 mRNA vaccine with standard number of dosing, 3) healthy subjects without any malignancy as controls, 4) the results of the studies included the seroconversion rate, antibody titer level, and adverse effects (AEs). The criteria for the studies to be excluded were: 1) a study other than a prospective cohort or clinical trial (i.e., cross-sectional studies, case reports, review articles, commentaries, or correspondence letters), 2) studies without available full-text articles; 3) single-arm studies or without healthy subjects as controls, 4) a cohort receiving stem cell transplant as active therapy.

### Data extraction and risk of bias assessment

The following data were extracted from each study: 1) first author and publication year; 2) study characteristics, such as study location, study design, and sample size; 3) characteristics of hematological malignancy cohorts, such as type of cancer, mean age, sex proportion, and type of active treatment received; 4) intervention details, such as type of vaccine, number of doses and follow up duration; 5) characteristics of healthy subject controls, such as control size, mean age, and sex proportion, and 6) outcomes presented. Primary outcomes were the seropositive rate, antibody titer level, and reported AEs, while other available outcomes were extracted as secondary outcomes. The risk of bias of each selected study was assessed using suitable appraisal tools. The Joanna Briggs Institute (JBI) checklist was used for nonrandomized intervention studies of intervention (e.g., cohort studies), while randomized clinical trials were evaluated using the Cochrane Risk of Bias 2.0 tool (18, 19). Any discrepancies among the five investigators (IR, IW, JR, LW, and SP) were resolved by discussion with an independent investigator (KW). The cutoff point values of the JBI checklist used to determine the level of bias of each study were the following: study with yes in equal or more than 70% of the questions labeled as low risk, 50%–69% of the questions labeled as moderate risk, and less than 50% of the questions labeled as high risk (20, 21).

### Data synthesis and statistical analysis

Quantitative analysis was conducted to calculate the cumulative outcome of positive seropositivity of mRNA vaccines in patients with hematological malignancies. A total of 15 studies were included in the quantitative analysis. The analysis was performed using Review Manager 5.4 (Cochrane). The analysis was based on dichotomous data with Mantel–Haenszel statistical method with random effects analysis model. The effect measures used on the analysis were risk ratio (RR) on the seropositivity rate comparison between the hematological malignancies group and its control group counterpart. The comparison was conducted on the first dose and second dose of vaccine, as well as the comparison between active treatment and non-active treatment. Subgroup analyses were used to explore cumulative outcomes of each hematological cancer type. The analyses were set on 95% confidence intervals (CI) with heterogeneity analysis using I^2^ as the outcome. The I^2^ cutoff of <50% indicated no significant heterogeneity, 50%–70% indicated considerable heterogeneity, 70%–90% high heterogeneity, and >90% indicated extremely high heterogeneity (22). The cumulative results were then extrapolated into Forest plots to describe the cumulative RR of the efficacy of the mRNA vaccine.

## Results

### Study selection

**Figure 1:**
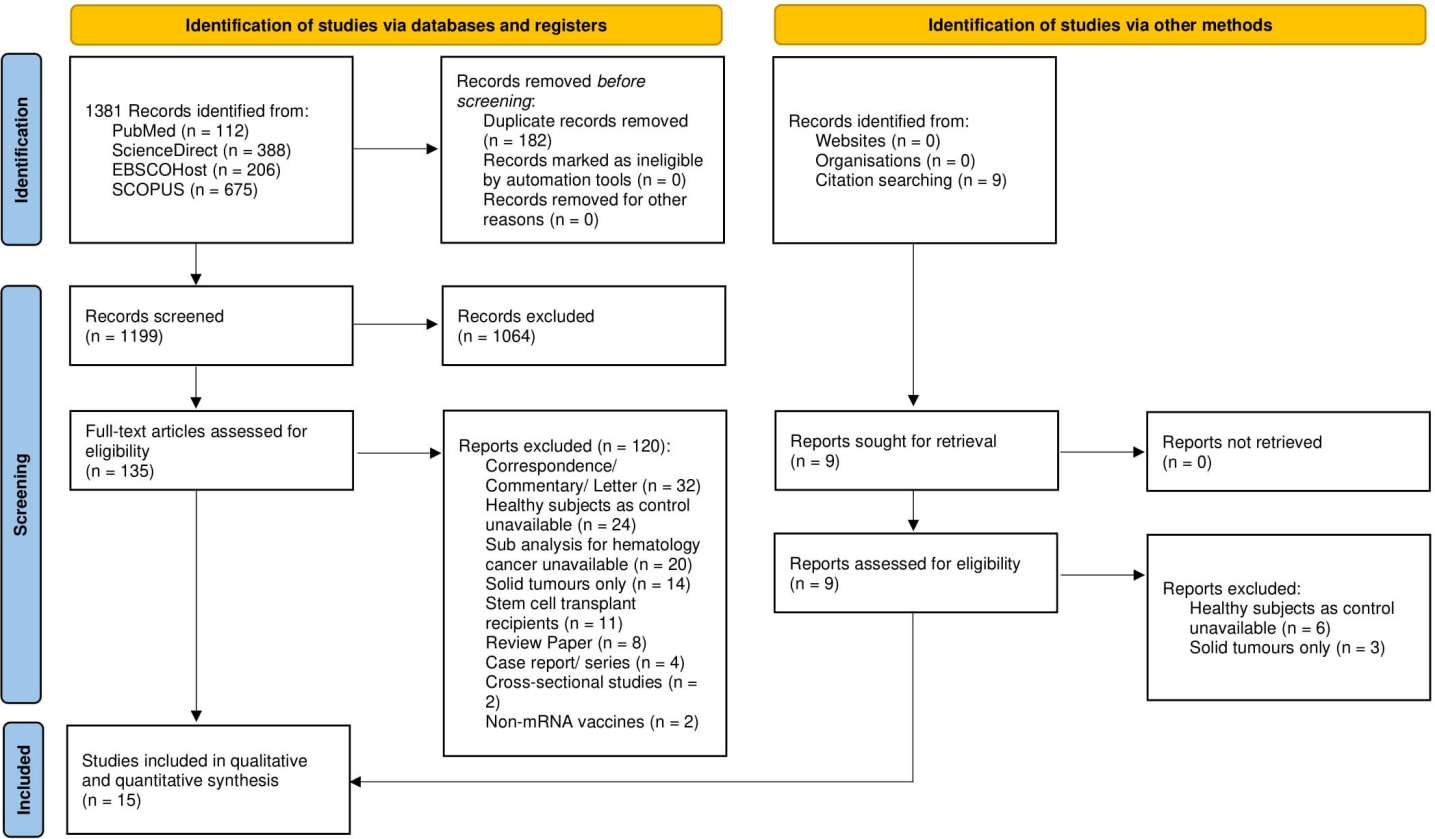
PRISMA flow diagram for systematic review and meta-analysis.

Searching the literature in four databases retrieved 1,381 articles, 182 of which were removed due to duplication. The titles and abstracts of 1,199 papers were selected and 1,064 papers were excluded due to irrelevancy. Full text reviews were omitted leaving 135 papers, and 120 papers were removed based on the exclusion criteria. Nine relevant studies were obtained through manual search, although they were later excluded based on the exclusion criteria. The remaining 15 papers were included in qualitative and quantitative synthesis. The summary of the study selection is presented in the PRISMA diagram (see **Figure 1**).

### Study characteristics and risk of bias

The 15 included studies were published between 2021 and 2022 in various countries and were used for the analysis. Most included studies were cohort prospective in design, except for one study which was an open-label, nonrandomized prospective clinical trial. A total of 2,055 patients with hematological malignancies were included of which 58.7% were men, with a median age range between 51 and 73 years. The control groups consisted of 1,105 healthy subjects and only 11 studies mentioned a detailed description of their age and sex proportion. Among the remaining studies, one matched age and sex, one matched age only, one matched sex only, and one did not match age and sex to the cohort with hematological malignancies. There were 5 studies that included multiple hematological malignancies in their cohorts and the other 10 studies included only a specific type of hematological malignancies. All the included studies had patients in different stages of the disease (i.e., active, chronic phase, remission, or relapse) and different stages of treatment (i.e., treatment naive, actively receiving various types of therapy, or refractory). Two doses of the BNT162b2 mRNA vaccine (Pfizer-Bio NTech) were used in all studies, except for a few studies (i.e., 3 studies with ChAdOx1 nCoV-19/AZD1222/Oxford-AstraZeneca vaccine and 3 studies with the mRNA-1273/Moderna vaccine). The duration of follow-up was also different between studies, ranging from 14 days to 72 days from the second dose. **Table 2** summarizes the baseline characteristics for all 15 studies included in this systematic review and meta-analysis.

**Table 2:** Population characteristics of the included studies.

Authors	Year Published	Study Location	Type of Study	Population						Intervention		Controls			
				Sample size	Median Age (range)	Sex	Type of Cancer	Active Treatment	Type of Vaccine	Number of Dosage	Follow Up Duration	Subjects	Sample Size	Median Age (range)	Sex Proportion
Parry et al. (23)	2021	Birmingham, United Kingdom	Cohort Prospective	299	69 (63-74)	Male 53% (159/299)	CLL or SLL	On BTKi (60); On venetoclax (6)	Pfizer (154); AstraZeneca (145)	2 doses	14 weeks (2 weeks after 2nd dose)	Healthy local participants	93	Age-matched controls	N/A
Tzarfati et al. (24)	2021	Be’er Ya’akov, Israel	Cohort Prospective	315	71 (61-78)	Male 56% (176/315)	Aggressive NHL (51); Indolent NHL (40); HL (16); MM (53); CLL (34); Acute leukemia (15); MDS (16); MPN (68); CML (22)	Chemotherapy (10); Chemoimmunotherapy (28); Anti-CD20 (2); Other MoAb (3); PI (6); IMIDs (12); BCR-ABL TKI (20); BCL2 inhibitor (4); JAK2 inhibitor (12); BTK inhibitor (5); PI/IMID/MoAb combination (20); others (40)	BNT162b2	2 doses	60 days	Healthy local participants	108	69 (58-74)	Male 44% (47/108)
Claudiani et al. (25)	2021	United Kingdom	Cohort Prospective	54	51.2	Males 51.9% (28/54)	CML in chronic phase, current treatment with TKI and in at least complete cytogenetic remission	TKI; 76% patients were receiving 2nd/3rd gen or newer TKI (dasatinib, nilotinib, bosutinib, ponatinib, and asciminib)	BNT162b2 or ChAdOx1 nCov-19 (Oxford–AstraZeneca) vaccine 6–12 weeks apart	2 doses	Up to day +49 +-7 after the second dose	Healthy Subjects	29	42.2	Male 62.1% (18/29)
Perry et al. (26)	2021	Tel Aviv, Israel	Cohort Prospective	149	64 (20-92)	Male 59% (88/149)	B-cell NHL, including diffuse large B-cell lymphoma + primary mediastinal B-cell lymphoma (69) and follicular lymphoma + marginal zone lymphoma (80)	Treatment naive (28), active treatment <6 mo from last anti CD20 therapy (39 combination R/Obi and 16 R monotherapy), completed treatment >6 mo (66)	BNT162b2 mRNA COVID-19 vaccine	2 doses, 21 days apart	14 to 21 days after the second dose (Serology tests) and 7 days after each of the 2 vaccine doses (AE)	Age-compatible, healthy volunteers, aged more than 18 years old	65	66 (25-83)	Male 45% (29/65)
Herishanu et al. (27)	2021	Tel Aviv, Israel	Cohort Prospective	167	71.0 (63.0 - 76.0)	Males 67.1% (112/167)	CLL or SLL	Naive (58), on therapy (75), off therapy in remission (24), off therapy in relapse (10); for treatment=BTKis ibrutinib or acalabrutinib (50), venetoclax +- anti-CD20 antibody (22), others (3)	BNT162b2 mRNA COVID-19 vaccine	2 Doses	2 to 3 weeks after administration of the second vaccine; 7 days after each vaccine dose (AE)	Age-matched AEs subjects	52	68	Matched with CLL cohort
Pimpinelli et al. (28)	2021	Rome, Italy	Cohort Prospective	92	MM cohort 73 (47-78); MPM cohort 70 (28-80)	MM male 54% (23/42); MPM male 52% (26/50)	42 patients with MM and 50 with MPM (Philadelphia-negative MPN n = 30 and CML n = 20)	MM (Proteasome inhibitor based 9, daratumumab based 14, imids based 19); MPM (hydroxycarbamide 20, TKI 20, ruxolitinib 6, interferon alpha 2, anagrelide 2)	BNT162b2 mRNA vaccination	2 doses 3 weeks apart	Up to 52 weeks from the first injection; 2 weeks after each injection (AE)	Elderly subjects aged over eighty not suffering from cancer	36	81 (79-87)	Male 50% (18/36)
Avivi et al. (29)	2021	Tel Aviv, Israel	Cohort Prospective	171	MM cohort 70 (28-94); SMM cohort 72 (49-79)	MM Male 57% (90/159); SMM male 50% (6/12)	MM (active 159; 34 were newly diagnosed and 79% were relapse or refractory patients) and SMM (12)	Active MM (159)=IMIDs 90, PI 73, DARA 72, IMID+PI 31, PRIOR HSCT 96 (60%) with median time since HSCT=36 (20-56) months; SMM (12)=N/A	BNT162b2 mRNA COVID19 vaccines	2 doses 21 days apart	14-21 days after the second vaccine	Age-compatible healthy volunteers	64	67 (41-84)	Male 42.1% (27/64)
Gavriatopoulou et al. (30)	2021	Athens, Greece	Cohort Prospective	106	73 (64-81)	Male 43% (46/106)	Waldenstrom Macroglobulinemia	Rituximab-ibrutinib (n=16), BTKi monotherapy (n=16), rituximab (n=1)	BNT162b2 (84.9%) and AZD1222 (15.1%) vs. BNT162b2 (82.1%) and AZD1222 (17.9%)	1 for AZD1222, 2 for BNT162b2	50 days	Above 60 years old	212	66 (62-82)	Male 46% (98/212)
Stampfer et al. (31)	2021	United States	Cohort Prospective	103	68 (35-88)	Male 59% (61/103)	96 with active MM and 7 with smoldering disease	Proteasome inhibitor (n=45), immunomodulatory agents (n-39), PI+IA (n=11), antibodies (n=19), alkylating agents (n=3), steroids (n=87)	BNT162b2 or mRNA-1273	2 dosages	baseline, 14-21 days post first and second dose	Healthy subjects were not known of immune status and therapy	31	69 (39-86)	Male 38.7% (12/31)
Bergman et al. (32)	2021	Stockholm, Sweden	Open label, non-randomized prospective clinical trial	90	<65 years (n=28)	Male 67% (60/90)	CLL	Indolent untreated (30), ongoing treatment with ibrutinib (30), previous ibrutinib treatment now in off phase (10), previous treatment with anti CD20 mAb (20)	BNT162b2	2 dosages	35 weeks after the second injection	Healthy individuals	90	<65 years (n=63)	Male 43.3% (39/90)
Monin et al. (33)	2021	London, United Kingdom	Cohort Prospective	56 for hematological malignancies (151 in total)	73 (64.5-79.5)	Male 52% (78/151); data for hematological malignancies only unavailable	Hematological (n=56); which included mature B-cell neoplasm (38/56), mature T-cell neoplasm (5/56), myeloid and acute leukemia (10/56)	Chemotherapy (n=2), targeted therapies (n=8), chemo/targeted therapies + immunotherapy (n=13), single agent MoAb (n=1), lenalidomide (n=1), radiotherapy (n=1)	BNT162b2	2 dosages	12 weeks after the first injection	Healthy Individuals mostly health care workers	54	40.5 (31.3-50)	Male 52% (28/54)
Malard et al. (34)	2021	Paris, France	Cohort Prospective	195	68.9 (21.5-91.7)	Male 60% (117/195)	Lymphoid malignancies (n=136; including, MM, NHL, HL, CLL, ALL, MGUS) and myeloid malignancies (n=59; including AML, MS, MPN)	Proteasome inhibitors, immunomodulatory drugs, anti-CD38 monoclonal antibodies, or steroids	BNT162b2	first and two doses	Day 28 and day 42 after first injection	Healthy Individuals, mostly health workers	30	Not matched	N/A
Tvito et al. (35)	2022	Jerusalem, Israel	Cohort Prospective	28	69 (54-94)	Male 71.4% (20/28)	Non-Hodgkin Lymphoma [Diffuse Large B-cell lymphoma (8); Follicular lymphoma (14); Marginal zone lymphoma (6)	Anti-CD20 mAbs (or completed therapy if still with-in 6 months); including Rituximab monotherapy (3); Rituximab maintenance (12); Bendamustine-rituximab (3); Bendamustine-obinutuzumab (2); R-CHOP (8)	Pfizer-BioNTech	2 doses (12 weeks apart)	72 days after first injection	Adult patients without NHL diagnosis (not specified)	28	50 (27-75)	Male 21.4% (6/28)
Cavanna et al. (15)	2022	Piacenza, Italy	Cohort Prospective	21 hematological malignancies (115 in total)	73 (72-76) - for all samples (including solid tumor)	Male 44.35% (51/115) - for all samples (including solid tumor)	Hematological malignancies (not classified)	Chemotherapy; immunotherapy; Anti-CD20; hormone therapy; etc. (not specific for hematological malignancies)	BNT162b2 mRNA vaccine (Pfizer–BioNTech) or the mRNA-1273 vaccine (Moderna)	2 doses	12 weeks (until 2nd dose)	Patients without malignancies, aged >70 years old	58	71 (70-74)	Male 39.66% (23/58)
Marasco et al. (36)	2022	Italy	Cohort Prospective	263	65	Male 53.3% (140/263)	59 patients (22·4%) had B-cell aggressive lymphoma, 111 (42·2%) B-cell indolent lymphoma or B-cell (CLL), 33 (12·6%) HL, 52 (19·8%) MM, and 8 (3%) T-cell lymphoma.	Chemotherapy (13.6%), Anti CD20 antibody plus chemotherapy (19.3%), IMIDs (9.9%), oral targeted therapy (8%), other therapies (13.4%)	mRNA-1273 n=243 (92.4%) and BNT162b2n=20 (7.6%).	two doses	4 weeks after first vaccine, 2 weeks after second dose	healthy health care workers	167	Matched age and sex	

CLL, chronic lymphocytic leukemia; ALL, acute lymphocytic leukemia; SLL, small lymphocytic leukemia; BTKi, Bruton’s tyrosine kinase inhibitors; HL, Hodgkin lymphoma; NHL, non-Hodgkin lymphoma; MM, multiple myeloma; SMM, smoldering multiple myeloma; MDS, myelodysplastic syndrome; MPN, myeloproliferative neoplasm; AML, acute myeloid leukemia; CML, chronic myeloid leukemia; MoAb, monoclonal antibody; PI, protease inhibitors; IMIDs, immunomodulatory drugs; TKI, tyrosine kinase inhibitors; BCL, B-cell lymphoma; JAK, Janus kinase; R/Obi, rituximab/obinutuzumab; R, rituximab; AE, adverse effects; MPM, myeloproliferative malignancies; DARA, daratumumab; HSCT, hematopoietic stem cell transplantation; HC, healthy control; MGUS, monoclonal gammopathy of undetermined significance; MS, multiple sclerosis; R-CHOP, rituximab cyclophosphamide hydroxydaunorubicin oncovin prednisone; N/A, not available.

Four articles did not clearly identify the confounders and five articles did not clearly mention their strategies to deal with the confounders. Two papers mentioned confounding factors but did not mention the strategies used to deal with them. Furthermore, in two papers the baseline data (especially those related to the outcome) of their cohort were not clearly defined. One paper showed different baseline antibody data between the population and control groups. Despite the limitations mentioned above, all the included studies had low levels of bias after being assessed using the JBI checklist. The detailed scoring based on the JBI checklist is provided in **Table 3**.

**Table 3:** Risk of bias assessment using the JBI checklist.

Name of Study		Q1	Q2		Q3	Q4		Q5	Q6		Q7	Q8	Q9	Q10	Q11		Level of Bias	
Parry et al. (23)		✓	✓		✓	?		?	✓		✓	✓	✓	N/A	✓		Low	
Tzarfati et al. (24)		✓	✓		✓	?		?	✓		✓	✓	✓	N/A	✓		Low	
Claudiani et al. (25)		✓	✓		✓	✓		✓	✓		✓	✓	✓	N/A	✓		Low	
Perry et al. (26)		✓	✓		✓	✓		✓	✓		✓	✓	✓	N/A	✓		Low	
Herishanu et al. (27)		✓	✓		✓	✓		✓	✓		✓	✓	✓	N/A	✓		Low	
Pimpinelli et al. (28)		✓	✓		✓	✓		✓	✓		✓	✓	✓	N/A	✓		Low	
Avivi et al. (29)		✓	✓		✓	✓		✓	✓		✓	✓	✓	N/A	✓		Low	
Gavriatopoulou et al. (30)		✓	✓		✓	✓		✓	✓		✓	✓	✓	N/A	✓		Low	
Stampfer et al. (31)		✓	✓		✓	✓		✓	✓		✓	✓	✓	N/A	✓		Low	
Bergman et al. (32)		✓	✓		✓	✓		?	?		✓	✓	✓	N/A	✓		Low	
Monin et al. (33)		✓	✓		✓	✓		X	X		✓	✓	✓	N/A	✓		Low	
Malard et al. (34)		✓	✓		✓	?		?	✓		✓	✓	✓	N/A	✓		Low	
Tvito et al. (35)		✓	✓		✓	?		?	✓		✓	✓	✓	N/A	?		Low	
Cavanna et al. (15)		✓	✓		✓	✓		✓	✓		✓	✓	✓	N/A	✓		Low	
Marasco et al. (36)		✓	✓		✓	✓		X	?		✓	✓	✓	N/A	✓		Low	

✓’ indicates yes, ‘**X**’ indicates no, and ‘?’ indicates unclear

JBI, Joanna Briggs Institute; N/A, not applicable.

### Study outcomes

#### Comparison of the rate of seroconversion after the COVID-19 mRNA vaccine

**Figure 2:**
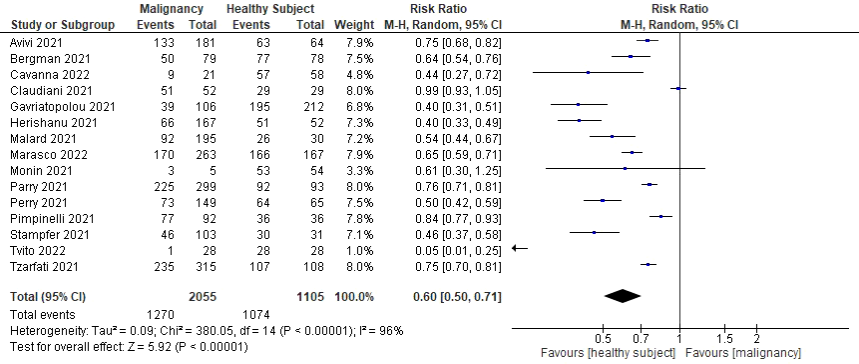
Forrest plot for the seroconversion rate after two doses of COVID-19 mRNA vaccination.

All 15 studies reported the seroconversion rate after SARS-CoV-2 mRNA vaccination, hence were all included in the qualitative and quantitative analysis (see **Table 4**). All cohorts with hematological malignancies showed lower rates of seroconversion after two doses of vaccination compared to healthy controls, although statistical significance was only mentioned in 8 studies. The pooled proportions of the seroconversion rate were 61.8% for the hematological malignancies cohort and 97.2% for the healthy controls. Meta-analysis of all included studies showed that after two doses of COVID-19 vaccination, only 60% of patients with hematological malignancy were seroconverted compared to healthy controls (RR=0.60; 95%CI 0.50–0.71; P<0.0001) as shown in **Figure 2**. There was high heterogeneity (I^2 =^ 96%), which was statistically significant.

**Table 4:** Outcomes of the included studies.

Name of Study	Method for Measurement and Cutoff for Seropositivity	Seropositive Rate	Antibody Levels	Adverse Effects	Other Reported Outcomes
Parry et al. (23)	Roche Elecsys anti-SARS-CoV-2 immunoassay to detect IgG against Spike protein (receptor binding domain) with seropositivity cutoff of ≥ 0.8 U/mL	Spike-specific antibody responses were detectable in 34% of CLL patients after one vaccine (29/86) compared to 94% in age-matched healthy donors. Antibody responses increased to 75% after the second vaccine (9/12), compared to 100% in healthy donors (59/59); patients with CLL received an equivalent proportionate antibody response after the second vaccine (although titers remained lower than those of the control group)	After the first dose: (0.4 vs. 41.6 U/mL, respectively; P<0.0001); antibody titers 104 times lower in the patient group compared to the AEs group. Second dose: 53 U/mL vs. 3900 U/mL; P<0.0001); Antibody titers 74-fold lower in CLL patients compared to healthy age-matched groups.	N/A	Previous natural SARS-CoV-2 infection exhibited stronger immune responses after COVID-19 vaccination in both the patient and control groups. Responses were found to be lower in groups on active therapy (especially on BTKi therapy) or who were due to start therapy soon. Serum concentrations of IgG, IgA, or IgM showed positive correlations with antibody response (but only for IgA were statistically significant).
Tzarfati et al. (24)	Liaison chemiluminescence immunoassay method to detect anti-S1 and S2 specific IgG with seropositivity cutoff of >12 AU/mL	The seropositivity in the cohort of hematological malignancies reached 235/315 (75%) vs. the AEs cohort 107/108 (99%) after two doses of vaccination (P<0.001)In a matched analysis (n=69 with paired age, sex, comorbidities, and time from vaccination to serology assay): 52/69 (75%) vs. 68/69 (99%) in AEs.	Median antibody titer of the cohort of hematologic malignancies 85 AU/mL (IQR 11-172) vs. AEs 157 AU/mL (IQR 130-221) with P<0.001. On matched analysis (n=69): 90 AU/mL (IQR 12-185) vs. 173 AU/mL (IQR 133-232) in AEs.	N/A	Seropositive patients had significantly higher absolute lymphocyte count (median [IQR]=1.5 [1.1–2.1] compared to 1 [0.6–1.88] × 103/μl; P<0.001), total globulin levels (29 [26–31] compared to 26 [22–30] g/L; P=0.003) and lower LDH (378 [316–444] compared to 427 [325–574] U/L; P=0.015) compared to seronegative patients. Patients who had never received treatment were more likely to obtain seropositivity, and patients who received treatment 0-6 months before vaccination had the lowest seropositivity rate (66%). The type of treatment also had a significant effect on the seropositivity rate.
Claudiani et al. (25)	Imperial double antigen binding ELISA method to detect IgG against Spike protein (receptor binding domain) with seropositivity cutoff of >1.8 BAU/mL	CML vs. HS; T1 = 48/50 (96%) vs. 25/26 (96.1%); T2 = 31/39 (79.5%, decreased P=0.019 vs. T1) vs. 25/27 (100%, P=0.99 vs. T1); T3 = 51/52 (98%) vs. 29/29 (100%); T4 = 45/46 vs. 26/26	Median CML vs. HS in Binding antibody units (BAU)/mL; T1 = 16.6 vs. 27.4 (P=0.8); T2 = 6.6 vs. 10 (P=0.2); T3 = 1867 vs. 2452 (P=0.29); T4 = 534.1 vs. 695.6 (P=0.25)	N/A	In univariate analysis, response status and TKI were not associated with anti-RBD levels in patients with CML (P=0.74 and 0.5 respectively); Age was inversely correlated with antibody responses only for HS (p 0.048); BNT162b2 was associated with higher anti-RBD responses (P<0.0001)
Perry et al. (23)	Roche Elecsys anti-SARS-CoV-2 immunoassay to detect IgG against Spike protein (receptor binding domain) with seropositivity cutoff of ≥ 0.8 U/mL	The Ab response to the COVID-19 vaccine was achieved in 73 of 149 (49%) patients with B-NHL included in our cohort, compared to 64 of 65 (98.5%) age-compatible AEs (P <.001).	Healthy controls had statistically significant higher Ab titers compared with the entire B-NHL patient cohort (mean titer, 1332 ± 1111 U/mL vs. 440 ± 1124 U/mL, respectively; P <.001), as well as when compared with each group of patients, separately (mean 1008 ± 1345 U/mL, 13.7 ± 98.5 U/mL, and 555 ± 1347 U/mL, in patients who were treatment-naïve, actively treated, or >6 months from last anti-CD20 Ab, respectively; P <.001).	Sixty of 118 evaluable patients (51%) reported AEs. The most common local AE reported in 44 (37.3%) patients was pain at the injection site. The most common systemic AE was fatigue (n=23; 19.5%), followed by muscle pain (n=11; 9.3%). Three (2.5%) patients reported transient lymph node enlargement. All AEs were mild and resolved spontaneously. There were no statistically significant differences in the types and severity of AEs between patients with B-NHL and AEs, except for pain at the injection site, which was reported to be more severe by patients with B-NHL.	Response rates in patients receiving an active anti-CD20 Ab–containing treatment regimen (chemoimmunotherapy or immune monotherapy) and in patients currently treated with R/Obi maintenance were 10.3% and 0%, respectively (P=.24), both significantly lower than in AEs (P <.001); Univariate analysis of the entire cohort of patients showed treatment status (current R/Obi treatment vs. therapy completed >6 months before vaccination vs. treatment-naïve; P <.001), ALC ≤.0.9 × 103/µL vs. ALC >.0.9 × 103/µL (P=.002), and any exposure to R/Obi (P <.001) since diagnosis to be significantly associated with lower response rates to the COVID-19 vaccine; Multivariate analysis, including age, ALC, disease type (i-B-NHL vs. a-B-NHL), and prior exposure to anti-CD20 Abs, confirmed that ALC ≤0.9 × 103/µL vs. higher ALC counts and any exposure to anti-CD20 therapy were independent predictors of negative serology
Herishanu et al. (27)	Roche Elecsys anti-SARS-CoV-2 immunoassay to detect IgG against Spike protein (receptor binding domain) with seropositivity cutoff of ≥ 0.8 U/mL	Antibody-mediated response in the CLL group (66/167 or 39.5%); analysis with 52 HS matched showed a significant reduction in the response rate 52% vs. 100% (adjusted OR 0.010, 95%CI 0.001-0.162; P<.001)	CLL median 0.824 U/mL (IQR 0.4-167.3 U/mL); 155 U/mL (IQR 7.6-490.3 U/mL) in responding patients with CLL; 1084 U/mL (IQR 128.9 -1879 U/mL) in HS with P<.001	The first dose=52 (31.1%) reported a mild local reaction and the second dose=56 (33.5%) reported a mild local reaction (pain at the injection site, local erythema or swelling) without statistically significant differences in local reaction rates between 2 dose; systemic 1st dose=21 (12.5%; weakness 11, headache 9, fever 4, muscle pain 3) and second dose=39 (23.4%) (weakness 14, fever 11, chills 10, headache 10, muscle pain 8) so more frequent after the second dose (P=.005) and all were mild; no significant correlation between local or systemic reactions and a positive serologic response to the vaccine; no correlation between AEs and active treatment; no correlation between AEs and active treatment	Univariate analysis variables were found to be significant: younger age, female, early stage of disease (Binet stage A), mutated IGHV, beta2-microglobulin <3.5 mg/L, untreated or off therapy >12 months, high levels of IgG, IgM and IgA levels; Multivariate analysis independent predictors of response=age <65 years OR 3.17, female sex OR 3.66, lack of active therapy OR 6.59, IgG levels >550 mg/dL OR 3.70, and IgM levels >40 mg/dL OR 2.92; Treatment naive had a higher response rate (55.2% vs. 16%, OR 0.16 95%CI 0.07-0.35) and a higher antibody level (median 1.7 U/mL vs. 0.4 U/mL, P<.001); no significant differences between patients receiving BTKis or venetoclax + anti-CD20 antibodies; high response rate (79.2%) and antibody levels (median 297.6 U/mL) were observed among 24 patients who completed treatment and maintained their response (CR/PR)
Pimpinelli et al. (28)	Liaison XL chemiluminescence immunoassay method to detect anti-S1 and S2 specific IgG with seropositivity cutoff of 15 AU/mL	TP1 (p vs. HC)=MM 9/42 (21.4%, P=0.005), MPM 26/50 (52.0%, P=1), HC 19/36 (52.8%); TP2 (p vs. HC)=MM 33/42 (78.6%, P=0.03), MPM 44/50 (88.0%, P=0.038), HC 36/36 (100%)	TP1 (p vs. HC)=HC 17.1 AU/mL, MM 7.5 AU/mL (P<.001), MPM 16.2 AU/mL (P=0.837); TP2 (p vs. HC)=HC 353.3 AU/mL, MM 106.7 AU/mL (P=0.003), MPM 172.9 AU/mL (P=0.049)	After the first dose=mild (20% pain, 10% tenderness, 1% headache, 3% malaise, 1% myalgia) and moderate (2% malaise); after the second dose=mild (13% pain, 7% tenderness, 3% fever, 2% headache, 1% malaise, 1% chills), moderate (3% pain, 1% tenderness, 1% fever, 1% myalgia, 1% chills), and severe (2% pain)	No sex effect (P=0.913); there was a significant trend to a lower response according to age increase in age (P<0.001) and for the disease cohort (both MM and MPM P<0.001); in MM cohort, patients on active treatment with proteasome inhibitors-based and IMID-based therapies (alone or in combo) without daratumumab had a higher likelihood of response compared to those on daratumumab (92.9% vs. 50%, P=0.003)
Avivi et al. (29)	Roche Elecsys anti-SARS-CoV-2 immunoassay to detect IgG against Spike protein (receptor binding domain) with seropositivity cutoff of ≥ 0.8 U/mL	MM=133/181 (78%); HC=63/64 (98%) P=0.00013; active MM=121/159 (76%); all patients with SMM had a serological response.	Median active MM=91 U/mL (0–4875); SMM 822 U/mL (5-2878); HC=992 U/mL (0.4-5,000)	For MM=any AEs 53%, pain injection site 44%, fatigue 15%, muscle pain 14%, headache 14%, fever 6%, dizziness 4%, rash 2%, chills 2%, lymphadenopathy 1%; for HC=any AEs 55%, pain injection site 43%, fatigue 19%, muscle pain 6%, headache 8%, fever 4%, arthralgia 2%	Univariate analysis of active (comparing responder vs. non)=older age (above 65), high risk cytogenetics, lower level of level of polyclonal globulins, lower lymphocyte count, advanced treatment line (second or third line), greater number of new drugs the patient was exposed to before vaccination and depth of response to anti-myeloma therapy at vaccination time were associated with a lower response rate; Daratumumab-containing regimens trended towards a lower response rate; Multivariate analysis revealed older age (P=0·009), exposure to 4 new antimyeloma drugs (P=0·02) and hypogammaglobulinemia (P=0·002) were associated with lower response rates.
Gavriatopoulou et al. (30)	GenScript ELISA cPass SARS-CoV-2 NAbs detection kit to detect NAbs with seropositivity cutoff of ≥ 30%	After the first dose: WM 34% (36/106) vs. HC 65% (138/212) with P<.001; After the second dose: WM 60.8% (45/74) vs. HC 92.5% (196/212) with P<.001	After the first dose: WM median Nab inhibition titer 20.5% (IQR 10-37%) vs. HC 39.8% (IQR 21.9-53.4%) with P<.001. After the second dose: WM 36% (IQR 18-78%) vs. HC 92% (IQR 70-96%) with P<.001	There were no differences between mild reactions (37% after the first dose vs. 38% after the second dose). Thirteen percent (after first dose) and 24% (after second dose) of patients developed systemic adverse reactions such as fatigue, fever, lymphadenopathy, muscle pain, arthralgia, headache.	BNT162b2 produced higher NAb compared to AZD1222 (median NAb 52% vs. 21.8% with P=.02). The asymptomatic subgroups had a higher median NAbs titer (52.9% vs. 44.3% for the symptomatic). Symptomatic patients who received Rituximab-based or Bruton tyrosine kinase as therapy showed suboptimal antibody response after vaccination.
Stampfer et al. (31)	Sino biological ELISA to detect IgG against Spike protein with seropositivity cutoff of ≥ 250 IU/mL	Using the 250 IU/mL cutoff, 45% of the MM patients responded, 22% partially (above 50 IU/mL), and 33% did not responded; all 7 patients with smoldering MM responded to vaccination; 2/31 HC had partial response and 29/21 fully responded	Active MM median IgG spike antibody 173.7 IU/mL (range 0.1 - 8215.9 IU/mL); Smoldering MM median 555.8 IU/mL (range 283.1 - 3162.9 IU/mL); HC median 893.6 IU/mL (range 116.7 - 6006.4 IU/mL)	N/A	Younger patients (<68 years) developed higher anti-spike IgG levels. Neither sex nor race were correlated with vaccine response. Patients with low lymphocyte counts had inferior responses. Patients who received steroids as treatment had reduced antibody levels. More advanced disease and worse disease status were indicative of a poorer response to mRNA vaccination.
Bergman et al. (32)	Roche Elecsys anti-SARS-CoV-2 immunoassay to detect IgG against Spike protein (receptor binding domain) with seropositivity cutoff of ≥ 0.8 U/mL	Lower in CLL (50/79) compared to controls (78/78) with P<0.01	N/A	More severe AEs in the CLL group (6 severe adverse reactions in 3 patients) than in the control group (n=0); all 6 were classified as moderate, were unlikely to be related to vaccination, and 5 of them resolved.	Ongoing treatment with mycophenolate mofetil and ibrutinib is noted to dampen the seroconversion process. Patients with a history of ibrutinib or anti-CD20 treatment had a higher seroconversion rate (55.6% and 88.9%, respectively).
Monin et al. (33)	Using the ELISA method to detect IgG against Spike protein with seropositivity cutoff of >70 EC50 dilution units OR EC50 was reached at 1:25 OR OD at 405 nm was 4 times higher than background	After the first dose for the hematological cancer cohort, the anti-SARS-CoV-2 IgG response was lower was lower (8/44 or 18% (95%CI 10-32) vs. HC (32/34 or 94% (95%CI 81-98) and after the second dose at day 21 for the hematological cancer cohort (3/5 or 60% (95%CI 23-88) vs. HC (12/12 or 100% (95%CI 76-100)	N/A	After the first dose, 65/140 cancer patients reported side effects (vs. 25/40 in the AEs group). After the second dose, 9/31 cancer patients reported side effects (vs. 9/16 in AEs). Injection-site pain was the most common local reaction (23/65 patients with cancer), others included injection-site erythema, swelling, fatigue, headaches, arthralgia, etc.)	Patients with hematological malignancies also showed a poorer response to T cell vaccine (measured as T cells producing IFN gamma or IL-2 producing T cells) compared to AEs and the cancer cohort sold (9/18 or 50% vs. 14/17 or 82% and 22/31 or 71%, respectively). There were no differences in the safety profiles between patients with solid and hematological cancer.
Malard et al. (34)	Using Abbott automated chemiluminescence assay method to detect IgG against Spike protein with seropositivity cutoff of ≥ 3100 UA/mL OR equal to NAbs ≥ 30%	After first dose: only 1.5% (3/195) patients seroconverted. After second dose: only 47% (91/196) of the patients achieved an anti-S IgG d42 level ≥3100 UA/mL after the two BNT162b2 inocula, compared to 87% (26/30) of AEs.	N/A	The most common were injection site pain (42.9%), fatigue (20.1%), and myalgia (10.4%). After the second injection of BNT162b2, 34.4% of the patients showed AEs (grade 1 to 2, 26%; grade 3, 8.4%; grade 4, 0%), with the most common types: injection site pain (grade 1 to 2, 23.4%; grade 3, 1.9%), fatigue (grade 1 to 2, 13%; grade 3, 5.8%), and myalgia (grade 1 to 2, 13%; grade 3, 3.9%)	Male sex, older patients, ongoing chemotherapy, and history of anti-B-cell treatment within the previous 12 months had significantly lower anti-S IgG after two doses of vaccination. Among patients without pathological B-cells, there was a strong positive correlation between the number of CD19+ B-cells with anti-S IgG antibody titers. T cell responses were detected in 53% (36/68) patients and were negatively affected by the active treatment received.
Tvito et al. (35)	Using the Abbott immunoassay method to detect IgG against the Spike protein with seropositivity cutoff of ≥ 150 UA/mL	Only one of 28 lymphoma patients (3.6%) developed a seropositive response, compared to 100% (28/28) of healthy volunteers.	N/A	N/A	Low levels of at least one immunoglobulin class were observed in 16 patients in the lymphoma group. CD19 + lymphocytes were not detected in 27 of 28 patients. All lymphoma patients treated with anti-CD20 mAb alone or in combination with chemotherapy did not exhibit a seropositive response after vaccination.
Cavanna et al. (15)	Using Liaison XL chemiluminescence immunoassay method to detect anti-S1 and S2 specific IgG with seropositivity cutoff of ≥ 15 AU/mL	Seropositivity in hematological malignancies: 9/21 (42.86%), whereas the control group was 100% (58/58)	The median IgG value at T1 was significantly higher in the seroconverted group (189 (IQR: 60–280) AU/mL vs. 3.8 (IQR: 3.80–5.55) AU/mL, p-value < 0.01)	N/A	There were no significant differences in seroconversion when comparing treatment status and received treatment (except for lower rates in patients treated with anti-CD20). Multivariate analysis showed a higher probability of seroconversion after vaccination (OR 3.30 with a 95% confidence interval (CI) of 1.23–8.87, p-value 0.02) for solid tumors compared to patients with hematological malignancies.
Marasco et al. (36)	Using Roche Elecsys anti-SARS-CoV-2 immunoassay to detect IgG against Spike protein (receptor binding domain) with seropositivity cutoff of ≥ 0.8 U/mL	From 263 subjects in the hematological malignancies cohort, 131 (49.8%; 95% CI 43.6%–56.0%) patients seroconverted four weeks after the first dose and 39 [14.8%; 95% confidence interval (CI) 11.0%–19.6%] two weeks after the second one, for a total of 170 (64.6%; 95% CI 58.5%–70.4%). Comparison with matched AEs also showed a lower rate of rate of rate of seroconversion in the cohort of cohort of cohort of hematological malignancies [64.1% (95%CI 56.3%-71.3%) vs. 99.4% (95%CI 96.7%-100%) with P<0.001].	The median antibody titer at two weeks after the second dose was 175 U/mL [interquartile range (IQR) 0.44–2.600]. Comparison with matched AEs showed lower antibody titers in the hematological malignancies cohort [median 207.5 U/mL (IQR 0.44-3,062) vs. 1,078 U/mL (IQR 643-1,841) with P<0.001].	N/A	Variables significantly associated with the lack of serological response included treatment in the last 12 months (especially for anti-CD20 antibody plus chemotherapy), type of malignancies, lymphopenia (<800 cell/uL), and low IgM levels. A total of 48 patients with malignancies on active treatment (out of 99 patients) showed the immune response (through assessing IFN-gamma, IL-2, TNF-alpha) two weeks after the second dose (vs. 99/99 in the matched AEs group).

Ig, immunoglobulin; CLL, chronic lymphocytic leukemia; BTKi, Bruton’s tyrosine kinase inhibitor; IQR, interquartile range; LDH, lactate dehydrogenase; ELISA, enzyme-linked immunosorbent assay; RBD, receptor binding domain; CML, chronic myeloid leukemia; HS, healthy subjects; HC, healthy control; TKI, tyrosine kinase inhibitors; AE, adverse effects; NHL, non-Hodgkin lymphoma; R/Obi, rituximab/obinutuzumab; ALC, absolute lymphocyte count; OR, odd ratio; CR, complete response; PR, partial response; TP, time point; MM, multiple myeloma; SMM, smoldering multiple myeloma; MPM, myeloproliferative malignancies; IGHV, immunoglobulin heavy chain variable region; NAb, neutralizing antibody titer; WM, Waldenstrom macroglobulinemia; OD, optical density; IFN, interferon; CI, confidence interval; IL, interleukin; TNF, tumor necrosis factor; N/A, not available.

**Figure 3:**
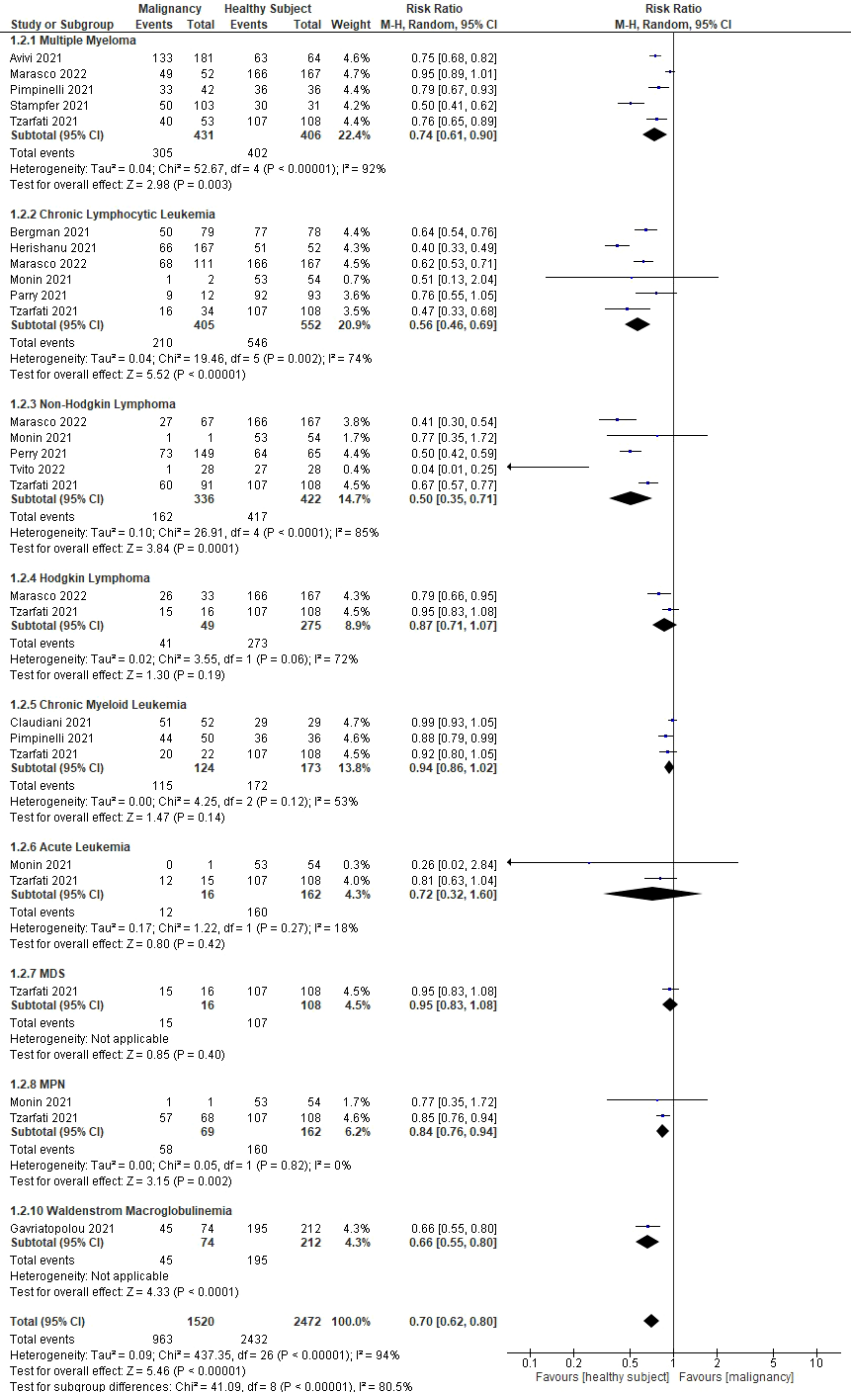
Sub-analyses showing the rate of seroconversion between different types of hematological malignancies.

**Figure 4:**
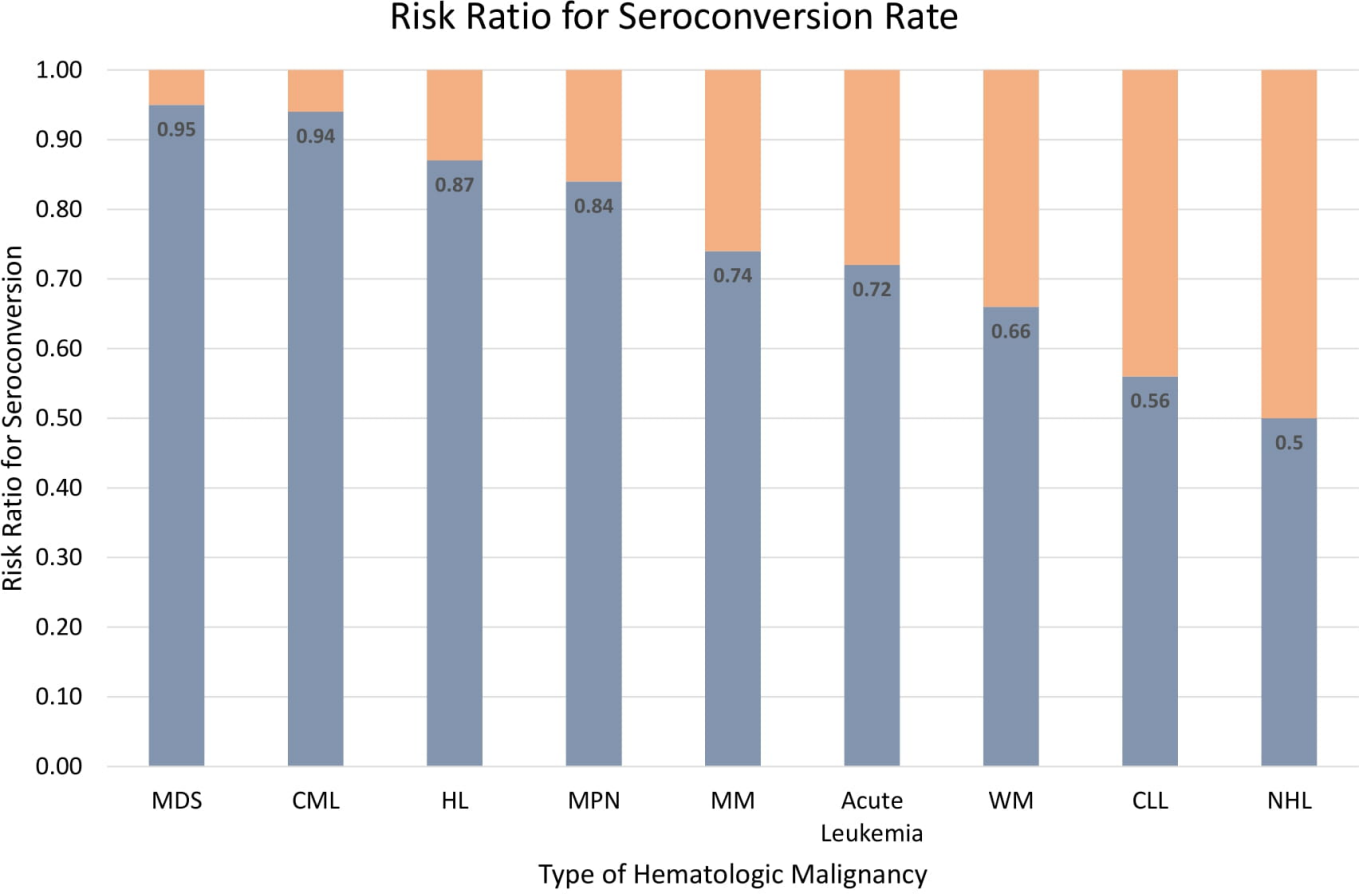
Risk ratio for seroconversion among different types of hematological malignancies.

An effort was made to minimize heterogeneity by performing a sub-analysis based on the type of hematological malignancy (see **Figure 3**). In 14 studies, nine different types of hematological malignancies were mentioned (Cavanna et al. (15) did not mention the type of malignancy). The results showed that patients with non-Hodgkin lymphoma (NHL) had the lowest rate of seroconversion (RR 0.5; 95%CI 0.35–0.71; P=0.0001), followed by chronic lymphocytic leukemia (CLL) (RR 0.56; 95%CI 0.46–0.69; P<0.0001), Waldenstrom macroglobulinemia (WM) (RR 0.5; 95%CI 0.35–0.71; P=0.0001), acute leukemia (RR 0.72; 95%CI 0.32–1.60; P=0.42), multiple myeloma (MM) (RR 0.74; 95%CI 0.61–0.90; P=0.003), myeloproliferative neoplasms (MPN) (RR 0.84; 95%CI 0.76–0.94; P=0.002), Hodgkin lymphoma (HL) (RR 0.87; 95%CI 0.71–1.07; P=0.19), chronic myeloid leukemia (CML) (RR 0.94; 95%CI 0.86–1.02; P=0.14), and myelodysplastic syndrome (MDS) (RR 0.95; 95%CI 0.83–1.08; P=0.40). **Figure 4** summarizes the risk ratio for each type of hematological malignancy. Significant heterogeneity (I^2^ above 35%) was found in five types of hematological malignancy (i.e., MM, CLL, NHL, HL, and CML). The sub-analysis of the acute leukemia and MPN groups did not show high heterogeneity, whereas it was not calculable in the MDS and WM groups.

**Figure 5:**
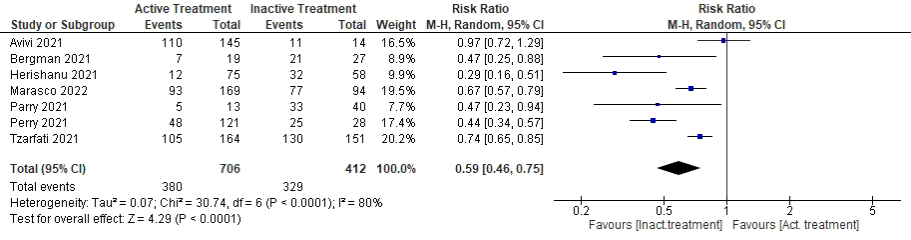
Sub-analysis of the seroconversion rate comparing active or inactive treatment.

Another sub-analysis was performed to compare the rate of seroconversion in patients with active treatments with patients who were treatment naive or had completed their treatments (see **Figure 5**). Data on treatment status were provided in 7 studies. Patients with active treatments had statistically significantly lower immunological responses toward the COVID-19 vaccination compared to patients not in active treatment (RR 0.59, 95%CI 0.46–0.75, P<0.0001). However, heterogeneity was also high in this sub-analysis (I^2 =^ 80%).

**Figure 6:**
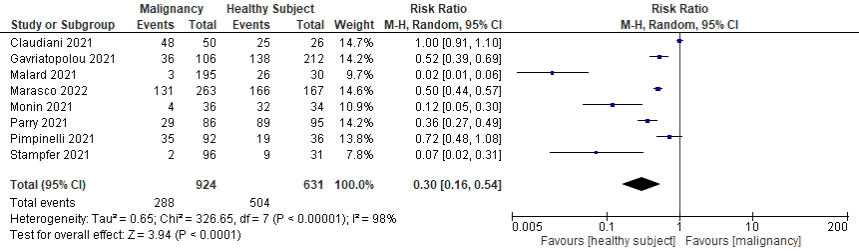
Sub-analysis of the seroconversion rate after receiving only the first dose of vaccine.

Our of 15 studies, eight reported the rate of seroconversion after the first dose and compared rates after the second dose. All studies showed a marked improvement in the rate of seroconversion after the second dose, although they were still lower than the healthy control group. A sub-analysis was performed to quantitatively measure RR when receiving only one dose of COVID-19 mRNA vaccine in relation to the rate of seroconversion (see **Figure 6** **)**. Receiving only one dose had a lower RR, which also indicated a lower rate of seroconversion, which was approximately half that of receiving two complete doses of the vaccine (RR 0.30; 95%CI 0.16–0.54; P<0.0001; I^2 =^ 98%).

#### Comparison of antibody titers after two doses of the COVID-19 mRNA vaccine

Eleven studies measured and reported antibody titers after two doses of SARS-CoV-2 mRNA vaccination and therefore were included in the qualitative analysis. Similar to the seroconversion rate, all studies found lower antibody titers in patients with hematological malignancies when compared with healthy controls (**Table 4** **)**. Herishanu et al. (27) showed that their CLL cohorts had more than a 1,000-fold lower median antibody titers and even the responding subgroup of CLL cohorts had a 7-fold lower median antibody titers than the healthy controls. According to other studies, Tzarfati et al. (24) (cohorts with various types of malignancy) and Gavriatopoulou et al. (30) (Waldenstrom macroglobulinemia cohorts) found lower antibody titers in cohorts of hematological malignancies by 2- and 3-fold, respectively. Marasco et al. (36) showed a larger gap of antibody titers (5 fold) between cohorts with various malignancies and healthy controls (207.5 U/mL vs. 1078 U/mL). Conversely, Claudiani et al. (25) found statistically insignificant differences in antibody titers between CML patients and healthy controls in their analysis.

Other studies also mentioned the median antibody titers between subgroups of their cohorts. Active treatment of patients in a study by Perry et al. (26) achieved lower antibodies when compared with treatment naive patients, although they were still lower compared to the healthy controls (13.7 U/mL, 1008 U/mL, and 1332 U/mL, respectively). Pimpinelli et al. (28) presented differences in antibody titers between different types of malignancies (MM 106.7 AU/mL vs. MPM 172.9 AU/mL). Different stages of the disease also had a significant impact on antibody titers. Stampfer et al. (31) and Avivi et al. (29) compared antibody titers for active MM and smoldering MM (early asymptomatic stage); cohorts with smoldering MM had 3 times higher antibody titers in the Stampfer et al. study and 9 times higher in the Avivi et al. study.

Some studies also measured antibody titers between the first and second dose of the vaccine, allowing comparisons to be made. MM patients in the study by Stampfer et al. (31) had a 15-fold increase in antibody titers after receiving the second dose. The study by Parry et al. (23) showed that the median antibody titers between CLL and healthy controls was 104 times lower after the first dose, which then decreased to 74 times lower after the second dose. Getting the second dose of vaccination also significantly increased antibody titers 2-fold in WM cohorts in the study by Gavriatopoulou et al. (30) and more than 100 times in CML cohorts in the study by Claudiani et al. (25).

#### Comparison of safety and adverse events after COVID-19 mRNA vaccine

Only 8 studies reported AEs observed after the SARS-CoV-2 mRNA vaccination. These studies showed that the COVID-19 the COVID-19 mRNA vaccines were safe for patients with hematological malignancies and healthy subjects. The analysis of Perry et al. (26) showed that there were no statistically significant differences in the types and severity of AEs between NHL patients and healthy controls. In contrast, in the studies by Avivi et al. (29) and Monin et al. (33), the incidence of AEs among healthy controls across all studies ranged from 20%–50%, most of which were mild localized reactions and resolved without any complication. Gavriatopoulou et al. (30) added that between the first and second doses, there was no difference in the occurrence of mild localized reactions (37% vs. 38%) but there was a difference in systemic adverse reactions (13% vs. 24%).

Pain at injection sites and local erythema or swelling were the most common mild AEs mentioned in these studies (26, 27, 33, 34). Commonly occurring systemic AEs were fatigue or weakness, muscle pain, headache, and fever (26, 27). Mild transient lymphadenopathy was found only in a small percentage of the subjects, about 1%–2.5%, cumulatively. However, in these studies there were also some moderate and severe AEs (26, 29). Pimpinelli et al. (28) observed the appearance of moderate AEs (e.g., malaise, fever, myalgia) in 9% and severe pain in 2% of their cohorts, especially after receiving the second dose of the vaccine. There were six moderate adverse reactions in the study by Bergman et al. (32), but they were unlikely to be related to vaccination and five of these resolved. Malard et al. (34) also reported the appearance of grade 3 AEs in 8.4% of their cohorts. Lastly, the analysis by Herishanu et al. (27) showed that the AEs were not correlated with a positive serologic response to the vaccine or with the treatment status.

## Discussion

COVID-19 has been the focus of researchers worldwide for the last 2 years. It has impacted the quality of life of the entire global population with its high transmissibility, morbidity, and mortality (37). Some populations have been shown to have higher susceptibility to this disease, including those with malignancies. Immunodeficiency in individuals with malignancies can be caused by the properties of the disease itself and by the effects of the radiation or chemotherapy (10). A study with 507,307 patients with COVID-19 showed that cancer patients who also received anticancer treatment within 3 months before the diagnosis of COVID-19 had an increased risk of hospitalization, admission to intensive care units, and death (38). Furthermore, COVID-19 vaccines have been developed and are considered essential to end the pandemic. Global efforts to create herd immunity are still in progress (39). Unfortunately, vulnerable individuals with malignancies seem to receive less protection from these vaccines. The meta-analysis by Sakuraba et al. (10) showed that cancer patients had a lower response to COVID-19 vaccinations compared to healthy individuals, especially in those with hematological cancer. Their finding is highly concerning, as hematological diseases are believed to have the highest level of immunosuppression and are associated with a 3- to 4-fold higher rate of severe COVID-19 disease, and even mortality (40–42). This systematic review and meta-analysis examining the efficacy and safety of the COVID-19 mRNA vaccine, specifically for people with hematological malignancies, is intended to help solve the problem.

In this systematic review, we identified 8 out of 15 studies reporting a seroconversion rate after administering the first dose of the COVID-19 mRNA vaccine (RR 0.30). However, the seroconversion rate was lower than that of the healthy control group. The antibody response improved significantly after the second dose (RR 0.60). Our findings also showed that patients with active treatments had a significantly lower immunological response than patients not receiving active treatment (treatment naive or had completed their treatment) (RR 0.59). The types of treatment could have a large impact on the response to vaccination, as all lymphoma cohorts that received anti-CD20 monoclonal antibodies in the study by Tvito et al. (35) did not show any serologic response. The seroconversion rate was also shown in MM, CLL, NHL, HL, CML, acute leukemia, MDS, MPN and WM. Receiving one dose of the COVID-19 mRNA vaccine showed a lower rate of seroconversion compared to two completed doses of the vaccine. Therefore, our findings recommend that patients with malignancy receive two doses of the COVID-19 mRNA vaccine. It can be speculated that disease-specific immunosuppression (such as hypoglobulinemia, B-cell dysfunction, and T-cell exhaustion) adds to treatment-related variables (27). In hematological malignancies such as CLL, the anti-SARS-CoV-2 antibody response to COVID-19 infection has already been reported to be low (67%); similar data have also been published for patients with MM convalescent to COVID-19, but data on anti-Ig for spike protein after COVID-19 vaccination have been limited so far (30, 31). Our data on vaccinated patients are consistent with three recently published observations in patients with CLL and MM (27, 28, 32).

This study emphasized lower antibody titers in hematological cancer patients compared to healthy populations. Hematological cancer patients were known to have two to seven times lower antibody titer levels, although a study could not conclude a significant difference (11). A breakdown of studies showed that patients who underwent active treatments also had lower antibody titer levels. Additionally, characteristics such as types of malignancy, stage of cancer, and number of vaccination doses also contribute to antibody titer levels in patients with hematological malignancy.

The results of this study were consistent with the findings of Teh et al. (11), which found a seropositivity rate of 37%–51% and 62%–65% after the first and second dose of the COVID-19 vaccine, respectively. The same study found that the lowest and highest conversion rates were found in CLL and acute leukemia with a 51% and a 93% rate, respectively. Furthermore, active treatment with targeted therapy and anti-CD20 monoclonal antibody showed the poorest immune response. The included studies also showed that patients receiving anti-CD20 monoclonal antibody-containing therapy (e.g., rituximab and obinutuzumab) had the lowest seroconversion rate (between 0% and 36%) (24, 26, 31). Another study suggested that the administration of rituximab could delay the immune response to COVID-19 after vaccination for a minimum of 6 months (43). Patients receiving a daratumumab-containing regimen (anti-CD38 monoclonal antibody) had higher seroconversion rate (range 58–69%) (28, 29). Regarding targeted therapies, patients receiving Bruton’s tyrosine kinase inhibitor (BTKi; for example, ibrutinib) or B-cell lymphoma-2 (BCL-2) inhibitor (e.g., venetoclax) had the lowest seroconversion rate (range 12.5%–52% and 13%–52%, respectively) (23, 24, 27, 32, 36). Therefore, considerations of active treatment should be made before administering the COVID-19 vaccine, especially in patients receiving targeted therapy and anti-CD20 monoclonal antibody.

Prophylactic agents are being developed to provide additional protection against COVID-19 in populations at high-risk for severe COVID-19 and vaccine nonresponders. Prophylactic agents available include the monoclonal antibody sotrovimab, tixagevimab-cilgavimab, casirivimab-imdevimab, and bamlanivimab-etesevimab (44). Intramuscular injection of tixagevimab-cilgavimab reduced the risk of symptomatic COVID-19 by 76.7% without any significant AE, as shown in the study by Levin et al. (45). The use of these prophylactic agents could become a solution to the low seroconversion rate in patients with hematological malignancies.

In regard to the safety issue, approximately half of all participants with hematological malignancy reported experiencing mild to moderate AEs. Mild AEs include pain at the injection site, swelling, and erythema followed by general fatigue and muscle pain or arthralgia. Of the moderate AES, most participants experienced fever, malaise, and lymphadenopathy. The hematological malignancy group reported significantly more AEs compared to the control group. However, there were no significant differences in AEs among treatment status, type of cancer, and disease status of patients. Similar results have also been reported by Teh et al. (11) and Fendler et al. (46), who reported mild AEs after vaccination in up to 40%–50% of patients.

To our knowledge, this study is by far the largest systematic review and meta-analysis of SARS-CoV-2 mRNA vaccination for cohorts specifically with hematological malignancies with 15 studies being included. Only studies with healthy subjects as control were included to clearly establish a cause and effect relationship of the independent variable (i.e., having hematological malignancies). Studies including patients with stem cell therapies were also excluded to reduce the chance of heterogeneity, because stem cell therapies are known to have immunomodulatory effects (47). Furthermore, the 15 included studies were found to have a low level of bias when evaluated using the JBI checklist.

Nonetheless, there are also some limitations to this study. This study was limited to a single type of vaccine (mRNA vaccine) to reduce heterogeneity, although there are approximately nine different vaccines currently available. Further research is encouraged to study the efficacy of the other types of vaccines in cohorts with hematological malignancies. Another limitation comes from the heterogeneity found in the meta-analysis. Several sub-analyses, conducted in an effort to explain heterogeneity found in the main meta-analysis, also showed significant heterogeneity and several factors could have caused the heterogeneity in this meta-analysis. Most studies evaluated the BNT 162b2 vaccine (Pfizer), although some also used mRNA-1273 (Moderna) and AZD1222 (AstraZeneca) vaccines, which could have produced different efficacy and safety profiles. The effect of different chemotherapy drugs and the disease status were not quantitatively measured due to the lack of data and the small number of sample sizes. Lastly, there were also differences in the methods used for measurement and cutoff for seropositivity. Although most studies used the Roche Elecsys anti-SARS-CoV-2 immunoassay method with a cutoff of 0.8 U/mL, some used different kits or methods and some even presented their data in different units (see **Table 4**). The data for control groups were incomplete in some studies; hence, some sub-analysis comparisons were conducted using the overall controls instead of the subgroup-specific controls.

## Conclusions

In this systematic review and meta-analysis of 15 studies, including 2,055 patients with hematological malignancies and 1,105 healthy controls, only 61.8% of hematological malignancies seroconverted after two doses of the SARS-CoV-2 vaccine mRNA compared to 97.2% for healthy controls (RR=0.60; 95%CI 0.50–0.71). Our sub-analyses showed that the type of malignancy affected the rate of seroconversion, because NHL patients had the lowest rate (RR 0.5; 95%CI 0.35–0.71), while patients with myelodysplastic syndrome had the highest rate (RR 0.95; 95%CI 0.83–1.08). Additionally, patients actively treated at the time of vaccination had a lower seroconversion rate (RR 0.59; 95%CI 0.46–0.75). Cohorts with hematological malignancies also showed inferior results in quantitative measurement of antibody titers compared to healthy controls. Fortunately, no increased AEs of vaccination were observed in the cohort of hematological malignancies, with most of the AEs being mild and eventually resolved. Thus, despite the increasing number of AEs due to the COVID-19 vaccination, the vaccine is still considerably safe for patients with hematological malignancies.

Undoubtedly, additional studies including larger sample sizes, a standardized measurement method and cutoff, and improved analyzing methods are needed. Observing increased seroconversion rates and antibody titers after the second dose, it will be intriguing to study the benefits of booster doses for specific groups of individuals, including those with hematological malignancies. Furthermore, we hope that the results of this meta-analysis could help policy makers devise better protection strategies for individuals with hematological malignancies, and even for patients who have already been vaccinated, through stricter health protocols, better personal hygiene practices, and further trials of prophylactic agents.

## Data availability statement

The original contributions presented in the study are included in the article/supplementary material. Further inquiries can be directed to the corresponding author.

## Author contributions

All authors have contributed substantially to the writing of this manuscript, with detailed contribution as follow: Conceptualization, Methodology, Supervision, and Project Administration by IR; Investigation, Writing – Original Draft Preparation, and Visualization by SP, LW, JT, and IW; Writing – Review and Editing by KW. All authors have read and agreed to the published version of the manuscript.

## Acknowledgments

The authors wish to extend our gratitude towards all authors of the included articles as the data source for this article.

## Conflict of interest

The authors declare that the research was conducted in the absence of any commercial or financial relationships that could be construed as a potential conflict of interest.

## Publisher’s note

All claims expressed in this article are solely those of the authors and do not necessarily represent those of their affiliated organizations, or those of the publisher, the editors and the reviewers. Any product that may be evaluated in this article, or claim that may be made by its manufacturer, is not guaranteed or endorsed by the publisher.
